# Assessing anger and irritability in children: psychometric evaluation and normative data for the German version of the PROMIS® Parent Proxy Anger Scale

**DOI:** 10.1007/s11136-021-03001-1

**Published:** 2021-09-29

**Authors:** Anne Kaman, Christiane Otto, Janine Devine, Michael Erhart, Manfred Döpfner, Tobias Banaschewski, Anja Görtz-Dorten, Charlotte Hanisch, Michael Kölch, Veit Roessner, Ulrike Ravens-Sieberer

**Affiliations:** 1 Department of Child and Adolescent Psychiatry, Psychotherapy, and Psychosomatics, University Medical Center Hamburg-Eppendorf, Hamburg, Germany; 2 Psychosomatic Clinic and Outpatient Center, Argora Clinic, Berlin, Germany; 3 Alice Salomon University of Applied Science, Berlin, Germany; 4 Apollon University of Applied Science, Bremen, Germany; 5 Department of Child and Adolescent Psychiatry, Psychosomatics and Psychotherapy, Faculty of Medicine and University Hospital Cologne, University of Cologne, Cologne, Germany; 6 School of Child and Adolescent Cognitive Behavior Therapy (AKiP), Faculty of Medicine and University Hospital Cologne, University of Cologne, Cologne, Germany; 7 Department of Child and Adolescent Psychiatry and Psychotherapy, Central Institute of Mental Health, Medical Faculty Mannheim, University of Heidelberg, Mannheim, Germany; 8 Department of Special Education and Rehabilitation, Faculty of Human Sciences, University of Cologne, Cologne, Germany; 9 Department of Child and Adolescent Psychiatry/Psychotherapy, University of Ulm, Ulm, Germany; 10 Department of Child and Adolescent Psychiatry, Neurology, Psychosomatics and Psychotherapy, Rostock University Medical Center, Rostock, Germany; 11 Department of Child and Adolescent Psychiatry and Psychotherapy, TU Dresden, Dresden, Germany

**Keywords:** Anger, Irritability, PROMIS, Children and adolescents, Psychometric evaluation

## Abstract

**Purpose:**

Anger and irritability are common and impairing symptoms in children. The PROMIS Anger scales assess self- and parent-reported irritable and angry mood over the past 7 days. The aim of this study was to evaluate the psychometric properties of the German version of the PROMIS Parent Proxy Short Form v1.0—Anger and to provide normative data.

**Methods:**

To evaluate the psychometric properties, data from the study ADOPT Epidemiology were used. In this study, the PROMIS Anger Scale was administered to a population-based sample of *n* = 8746 parents of children aged 8–12 years. Psychometric analyses were carried out including the investigation of distribution characteristics, factor structure, model fit, internal consistency, and congruent validity. Normative data were calculated as percentile ranks and T-scores.

**Results:**

The PROMIS Anger Scale demonstrated good psychometric properties, including satisfactory distribution characteristics, unidimensionality, good internal consistency as well as congruent validity. German normative data for the PROMIS Anger Scale are presented.

**Conclusion:**

Based on first psychometric analyses, the German version of the PROMIS Anger Scale can be recommended for use in research and practice; however, further investigations using clinical data are needed. The normative data will allow researchers and clinicians an interpretation of the test scores in future applications.

## Background

Anger is a negative emotional state that is elicited by interpersonal provocation or frustration and often manifests itself in verbal and/or physical aggressive behavior [1, 2]. Irritability is conceptualized as a low threshold for experiencing symptoms of negative affectivity such as anger in response to frustration. Thus, anger, frustration, aggression, and irritability are interrelated psychological constructs [3].

Anger and irritability are common and impairing symptoms in children and adolescents and are among the most common reasons for referrals to child and adolescent mental health services [3]. According to the Diagnostic and Statistical Manual of Mental Disorders, Fifth Edition (DSM-5), irritability is an associated symptom of numerous mental disorders, including attention-deficit/hyperactivity disorder (ADHD), oppositional defiant disorder (ODD), and disruptive mood dysregulation disorder (DMDD) [4]. Furthermore, irritability in childhood and youth often predicts anxiety and depressive disorders in adulthood [5, 6]. Since irritability can have serious psychosocial implications and can predict long-term adversities [7, 8], the reliable and valid assessment of anger and irritability is important in order to identify and treat affected children at an early stage.

In the assessment of psychological symptoms, patients’ reports on their symptoms, well-being, and functioning play a crucial role, as outcomes such as emotions and affects are best known to the patients themselves. In recent years, patient-reported outcomes (PROs) have become increasingly important in health care, clinical research, and evaluation studies [9, 10]. Against this background, the National Institute of Health (NIH) set up an initiative to develop and evaluate a set of accessible, flexible, and psychometrically sound item banks to measure a broad range of PROs—the Patient-Reported Outcome Measurement Information System (PROMIS®). Those measures aim to be more reliable, valid, and responsive compared to existing PROs and enable an efficient application in research and clinical settings [11, 12]. The PROMIS measures capture physical, mental, and social aspects of health and can be used in the general population as well as in individuals living with chronic conditions. The item banks allow for the assessment of PROs via customized short forms and computer adaptive tests (CATs) by item response theory (IRT) models [13, 14].

To assess patient-reported emotional distress, three PROMIS item banks have been developed following the methodology of item development adopted by the PROMIS initiative. This includes comprehensive literature searches to identify existing items, item classification and selection, qualitative item review and revision, focus groups with patients, cognitive interviews, and final item revision before field testing [15–18]. The calibrated item banks cover three domains of emotional distress—depression, anxiety, and anger. The PROMIS Anger item banks offer a dimensional assessment of irritable and angry mood, frustration as well as aggressive behavior over the past seven days. Besides the comprehensive item banks, the PROMIS Anger instruments are available as customized short forms and as CATs. Further, PROMIS provides Anger instruments for pediatric self-report (ages 8–17), adult self-report (ages 18 +), and parent proxy-report (children ages 5–17) [15]. Although self-report should be considered the standard in the assessment of PROs, there may be circumstances in which parent proxy-reports are required, e.g., in situations in which the child is cognitively impaired or too young to complete a questionnaire [19, 20]. Moreover, research has shown that prevalence estimates for externalizing problems that are based on self-report are generally lower compared to parent proxy-reported symptoms [21, 22]. Externalizing behavior problems such as anger and aggression can be better observed by parents and are less prone to dissimulation tendencies. Thus, parent proxy-reports are considered to be reliable and relevant sources of information.

The PROMIS initiative aims to implement PROMIS measures in epidemiological, clinical, and health care research across the world. The aims of this study were to evaluate the psychometric properties of the German version of the PROMIS Parent Proxy Short Form v1.0—Anger in a population-based sample—and to provide German normative data that will facilitate interpretation of the test scores in research and practice.

## Methods

### Study

Psychometric evaluation of the PROMIS Anger Scale was performed using data from the study ADOPT Epidemiology, which is part of the research consortium ADOPT (Affective Dysregulation – Optimizing Prevention and Treatment). In the study ADOPT Epidemiology, data on affective dysregulation and irritability in children were collected in a population-based sample across four German cities (Cologne, Dresden, Mannheim, and Ulm) over the course of 19 months (February 2018 to August 2019). Families with children aged 8–12 years were randomly selected from the residents' registration offices of the four cities. Potential participants were informed about the study and asked for their participation. Once their written informed consent was obtained, the parents were asked to complete a paper–pencil questionnaire. Alternatively, the participants had the opportunity to complete the questionnaire online or to answer the questions on the phone. Electronic data collection and management were supported using a secure, web-based application named REDCap [23]. The study ADOPT Epidemiology was approved by the ethics committee of the General Medical Council Hamburg and the commissioner for data protection from the University Hospital Cologne. For further details concerning the design and methods of the research consortium ADOPT and the sub-project ADOPT Epidemiology, see Döpfner et al. [24] and Otto et al. [25].

### Participants

Of the *N* = 79,015 potential participants contacted within the population-based screening of the study ADOPT Epidemiology, *n* = 10,288 (13.7%) parents agreed to participate. Participants were included in the present analyses if (i) their child was between 8 and 12 years of age at the time of participation and (ii) they answered at least one of the items of the PROMIS Anger Scale. The final sample under analysis included *n* = 8746 parents of children aged 8–12 years.

### Measures

#### Sociodemographic variables

Age (in years) and gender of the child as well as the education of the parents were assessed. Parental education, an indicator of the socioeconomic status (SES), was assessed by two items asking for the highest academic and vocational qualification of both parents. Children were assigned the maximum point score their parents provided (depending on which parent had a higher level of education). Levels of education were operationalized based on the international ‘Comparative Analysis of Social Mobility in Industrial Nations’ (CASMIN) classification of education [26]. This classification differentiates nine categorical levels of education based on distinct combinations of academic and vocational qualifications. Based on these combinations, a categorization into parents with low (primary), medium (secondary), and high (tertiary) education was performed.

#### PROMIS Anger Scale

Parents completed the German translation of the PROMIS Anger Scale [15, 27]. The scale consists of five items covering parent-reported irritable and angry mood of the child over the past seven days (e.g., ‘My child felt mad’). Items were rated on a 5-point response scale ranging from 0 (never) to 4 (almost always), with higher scores indicating more severe symptoms. Using the scoring tables provided in the PROMIS Anger Scoring Manual, the total score of the scale was calculated and translated into a standardized T-score, which allows an interpretation of a person’s anger symptoms compared to other individuals in the reference population. A score of 50 represents the mean T-score of the US general population (parents with 5- to 17-year-old children) with a standard deviation of 10. Symptom scores of 0.5, 1.0, and 2.0 standard deviations above the mean indicate mild, moderate, and severe symptoms.

#### Symptoms of affective dysregulation

For the assessment of congruent validity, symptoms of affective dysregulation in children were measured using the Screening Tool for Affective Dysregulation in Children (DADYS-Screen) [25]. The parent-reported screening tool includes 12 items focusing on symptoms of persistent irritability and severe temper outbursts in children (e.g., ‘Often loses temper’ or ‘Gets angry frequently’). Items were offered with a 4-point response scale ranging from 0 (not at all true) to 3 (completely true). The raw sum scores ranged from 0 to 36.

### Data analysis

The psychometric properties of the PROMIS Anger Scale were examined following the recommendations and guidelines of the PROMIS initiative [28]. First, common item characteristics including mean (*M*), standard deviation (SD), response frequencies, proportion of missing values, item difficulties, item-total correlations, and inter-item correlations were calculated. At the scale level, distribution characteristics including range, mean, standard deviation, skewness, and kurtosis of the raw sum score as well as US T-scores were examined.

A confirmatory factor analysis (CFA) was conducted to evaluate unidimensionality of the PROMIS Anger Scale using a weighted least squares means and variance adjusted (WLSMV) estimator. To examine model fit, the comparative fit index (CFI), Tucker–Lewis index (TLI), root mean square error of approximation (RMSEA) with 90% confidence interval (CI), and the standardized root mean squared residual (SRMR) were taken into account. CFI and TLI values ≥ 0.95 [29, 30], RMSEA values ≤ 0.06 [29], and SRMR values ≤ 0.08 [29] indicate a good model fit.

Internal consistency was examined as a measure of reliability via Cronbach’s α coefficient with values above 0.70 indicating acceptable reliability [31]. As a measure of construct validity, congruent validity was assessed by examining the correlation between the PROMIS Anger Scale and the DADYS-Screen. We calculated the Pearson correlation coefficient (*r*), expecting both scales to be strongly positively correlated (*r* > 0.50).

Finally, normative data for the German version of the PROMIS Anger Scale were calculated. To assess the need for age- and gender-specific normative data, effects of age and gender were examined using analyses of variance (ANOVA). Following this, a rank-based transformation [32, 33] was performed due to the non-normal distribution of the PROMIS Anger test scores. Based on the cumulative frequencies of the raw scores, percentile ranks (PR) were calculated and transformed into normalized z-scores. These z-scores were then transformed into standardized T-scores with a mean of 50 and a standard deviation of 10. Mplus 8 [34] was used for CFA, and all other analyses were conducted using IBM SPSS Version 27 [35].

## Results

The analyzed sample including *n* = 8746 parents of children and adolescents aged 8–12 years is described in Table 1. About half of the investigated children and adolescents were female (48.7%), and the mean age was 10 years (SD = 1.38). The questionnaire was answered predominantly by mothers (76.8%) of the participating children; in 17.3% of the cases, fathers responded; for 5% of the children, both mothers and fathers answered the survey together; and for 0.9% of the children, other relatives (e.g., step-, foster- or grandparents) provided proxy-reports. Most of the parents were highly educated (69.4%), 27.3% had a medium and 3.3% a low educational level.

**Table 1:** Description of the analyzed sample

	*n* = 8746		
	*n*	*%*	*M *(SD)
Gender of the child			
Male	4489	51.3	
Female	4257	48.7	
Age of the child			10.00 (1.38)
Respondent			
Mother	6714	76.8	
Father	1510	17.3	
Mother and father	440	5.0	
Others	82	0.9	
Parental education^a^			
Low	281	3.3	
Medium	2316	27.3	
High	5886	69.4	

^a^Missing values were given for *n* = 263 for parental education

### Descriptive statistics

The item characteristics of the PROMIS Anger Scale are shown in Table 2. Item-level means ranged from 0.38 (‘My child was so angry he/she felt like throwing something’) to 1.56 (‘My child felt upset’). Considering the threshold of 15% of respondents scoring at the lowest possible category [36], floor effects were observed for all items except item 4 (‘My child felt upset’). The proportion of missing values was very low, ranging from 0.1 to 0.4% per item. Item difficulties ranged from *p*_*i*_ = 0.10–0.39, and corrected item-total correlations ranged from *r*_it_ = 0.65–0.81. As displayed in Table 3, medium to strong inter-item correlations were found with correlation coefficients ranging from *r* = 0.47–0.73. The distribution characteristics of the PROMIS Anger Scale are shown in Table 4. The raw sum scores ranged from 0 to 20 (*M* = 4.37, SD = 3.55), and the standardized US T-scores ranged from 29.0 to 85.0 (*M* = 44.38, SD = 10.48). The scale had a positively skewed distribution, supporting the results of the item analysis. The low kurtosis indicated a platykurtic distribution of the scale, characterized by a lower peak and shorter tails compared to the normal distribution.

**Table 2:** Item-level descriptive statistics of the PROMIS Anger Scale translated to German

		Mean (SD)	Response frequencies (%)					Missing values (%)	*p*_i_	*r*_it_
			Never	Rarely	Sometimes	Often	Almost always			
1	My child felt mad	1.20 (0.88)	23.0	41.3	28.6	6.6	0.4	0.1	.30	.78
2	My child was so angry he/she felt like yelling at somebody	0.81 (0.98)	51.0	24.6	18.0	4.9	1.3	0.2	.20	.81
3	My child was so angry he/she felt like throwing something	0.38 (0.76)	75.5	14.1	7.8	2.0	0.5	0.1	.10	.66
4	My child felt upset	1.56 (0.94)	13.1	33.4	38.9	12.2	2.0	0.4	.39	.70
5	When my child got mad, he/she stayed mad	0.42 (0.73)	69.6	20.7	7.6	1.7	0.3	0.2	.11	.65

*p*_i_ = item difficulty; *r*_it_ = corrected item-total correlation

**Table 3:** Inter-item correlations of the PROMIS Anger Scale translated to German

		1	2	3	4	5
1	My child felt mad	–				
2	My child was so angry he/she felt like yelling at somebody	.73***	–			
3	My child was so angry he/she felt like throwing something	.53***	.60***	–		
4	My child felt upset	.68***	.63***	.47***	–	
5	When my child got mad, he/she stayed mad	.54***	.55***	.51***	.51***	–

Inter-item correlations are presented by Spearman rank correlations; *** *p* < .001

**Table 4:** Scale-level descriptive statistics of the PROMIS Anger Scale translated to German

	Range	*M*	SD	*S*	*K*
PROMIS Anger Scale raw sum scores	0–20	4.37	3.55	1.07	0.85
PROMIS Anger Scale T-scores^a^	29.0–85.0	44.38	10.48	0.70	0.09

*M* mean, *SD* standard deviation*, S* skewness, *K* kurtosis

^a^Based on US reference population

### Dimensionality

The descriptive fit indices resulting from the CFA pointed to a good model fit (RMSEA = 0.066, 90% CI 0.058–0.074, SRMR = 0.018, CFI = 0.998, TLI = 0.996, *χ*^2^ (5, *N* = 8,746) = 194.365, *p* < 0.001). The standardized factor loadings ranged from 0.79 to 0.93. Residual correlations among items were very low (between − 0.03 and 0.04). Thus, findings indicated that the 5-item PROMIS Anger Scale can be considered sufficiently unidimensional, confirming the factorial validity of the scale.

### Reliability and congruent validity

The internal consistency of the PROMIS Anger Scale in the present study was good with Cronbach’s *α* = 0.88. Further, in support of congruent validity, a strong positive correlation was found between the PROMIS Anger Scale and the DADYS-Screen (*r* = 0.78; *p* < 0.001).

### Normative data

Percentile ranks and German T-scores for the total sample were given as normative data (see Table 5). Although there were significant effects of age (*F*(4, 8671) = 10.122, *p* < 0.001, *ƞ*^2^ = 0.005) and gender (*F*(1, 8674) = 38.048, *p* < 0.001, *ƞ*^2^ = 0.006), they did not reach the lower limit of practical significance of 1% (eta squared as a measure of effect size was below 0.01). Thus, no age- and gender-specific normative data were given.

**Table 5:** Normative data (percentile ranks and German T-scores) for the PROMIS Anger Scale in 8- to 12-year-old children

Raw score	Percentile rank	T-score
0	9.07	28.4
1	21.54	39.4
2	37.93	44.5
3	51.02	48.3
4	61.56	51.7
5	68.94	54.0
6	75.53	55.9
7	81.21	57.9
8	86.14	59.9
9	90.34	62.0
10	93.20	64.0
11	95.03	65.7
12	96.73	67.5
13	97.82	69.3
14	98.84	71.4
15	99.35	73.7
16	99.61	75.7
17	99.75	77.3
18	99.91	79.0
19	99.94	80.0
20	100.00	80.0

## Discussion

The aim of the present study was to investigate the psychometric properties of the German version of the PROMIS Anger Scale in a population-based sample of parents with children aged 8–12 years. Overall, the German translation of the PROMIS Anger Scale demonstrated good psychometric properties, including unidimensionality, good fit statistics, good internal consistency, and congruent validity. The normative data will allow German clinicians and researchers an interpretation of the test scores in clinical practice and future studies.

The descriptive analyses showed very few missing values, indicating a good acceptability of the items. In line with Pilkonis et al. [15] who examined the distribution characteristics for the original English version of the PROMIS Anger Scale, we detected rather low item difficulties as well as floor effects for most of the items, which could indicate limited content validity and reduced variability in the data. Only very few parents reported that their children exhibited irritable or angry mood *often* or *almost always*. As a consequence, while the measure allows good differentiation between respondents with stronger irritability, healthy children and children with mild irritability cannot be differentiated very well by the PROMIS Anger Scale. The positively skewed distribution of the scale can be attributed to the fact that we conducted a symptom screening in a population-based sample, in which the prevalence of angry mood and aggressive behavior is generally lower compared to prevalences in clinical samples. Future studies could apply IRT methods to deal with the skewed distribution of the scale.

The confirmatory factor analysis showed that the hypothesized unidimensional model structure fits the data reasonably well. Model fit indices were well above popular rules of thumb and the standardized factor loadings were high. Thus, the factorial validity of the PROMIS Anger scale was confirmed.

The internal consistency of the scale was good, indicating that the PROMIS Anger Scale is a reliable instrument to measure anger and irritability in children. Further, our results provide support for the construct validity of the PROMIS Anger Scale, which showed congruent validity with a measure on affective dysregulation in children (DADYS-Screen). Future studies may also test the discriminant validity using, for example, measures of psychological functioning, positive affect or global health. For the original English version of the PROMIS Anger Scale, Pilkonis et al. [15] found evidence of discriminant validity using PROMIS Global Health items as divergent measure (*r* = 0.40).

Although we found significant effects of age and gender on parent-reported symptoms of anger, these effects did not reach the lower limit of clinical relevance of 1%. This is in line with results by Humphreys et al. [37], who found no gender differences in levels of irritability in a community sample of children aged 9–13 years. However, it could be that practically relevant gender differences first emerge during adolescence and become more pronounced during puberty. Future studies are needed to examine this relationship in more detail.

The mean symptom severity (T-score = 44.36) in our sample was considerably lower compared to the mean in the US reference population (T-score = 50). This finding is in line with a cross-cultural study that found that US parents reported more externalizing problems for their children compared to German parents [38]. Mean scores can differ among countries because of cultural differences or as a result of the translation. Considerable mean differences between countries limit the comparability of PROs and cross-national research. Future studies should conduct differential item functioning (DIF) analyses to examine if participants from different countries with the same level of anger and irritability respond differently to the items. Furthermore, more research on alternative calibration and centering approaches is needed to facilitate the interpretation of PROMIS T-scores across countries and populations [39]. The reference values available on the PROMIS website are based on a sample of parents with children aged 5–17 years of the US general population that matches the distribution of age, gender, race, and education in the 2000 US Census [40]. As our sample consisted of parents with children aged 8–12 years, it is not fully comparable to the US reference sample. Moreover, less educated parents were underrepresented in our sample. Therefore, the German normative values provided in the present study apply to this reference group only and described findings should not be generalized to children and adolescents outside this age range. For the German normative values provided, a T-score of 50 represents the average score of parent-reported anger symptoms among children aged 8–12 years in the German general population. The calculated percentile ranks and T-scores have the advantage that they can be used in the case of a non-normal distribution as they are based not on a linear, but on a rank-based transformation of the raw scores [32, 33].

Our study has the following limitations: First, our analyses were based on a population-based sample of parents with children aged 8–12 years. Thus, findings should not be generalized to children and adolescents outside this age range. Second, we had no access to sociodemographic or health-related information of the non-participating parents. The fact that participants with a low level of education were underrepresented in our sample may indicate that non-response was associated with parental education. This should be taken into account when interpreting the results, as research has shown that externalizing problems are more common among children with a lower SES [41]. Further, it should be noted that our translation and psychometric testing was based on the PROMIS Anger Scale Short Form v1.0. However, this version is highly comparable to the recently developed Short Form v2.0 because the underlying items and response options are identical. The only difference lies in the coding of the response scales ranging from 0 (never) to 4 (almost always) in version 1.0 (raw scores from 0 to 20) and 1 (never) to 5 (almost always) in version 2.0 (raw scores from 5 to 25), respectively.

This study has several strengths. Psychometric analyses were based on a very large population-based sample and were conducted in accordance with the recommendations for psychometric evaluation after translation of PROMIS instruments. Further, data were collected by means of an online survey, paper–pencil questionnaire, or telephone interview, depending on what the participants preferred, in order to minimize barriers and increase willingness to participate. Lastly, the country-specific normative data can help facilitate an interpretation of the test scores for German researchers and clinicians in future applications of the PROMIS Anger Scale in research and practice.

Overall, our findings provide evidence of the internal consistency, congruent validity, and unidimensionality of the German version of the PROMIS Anger Scale as a measure of anger and irritability in children. Future studies may wish to undertake further psychometric analyses, including the investigation of discriminant validity and differential item functioning as well as further investigations in a clinical sample using methods based on IRT. On the basis of our results, the German version of the PROMIS Anger Scale can be recommended for use in future studies and clinical applications.

## Data Availability

The data that support the findings of this study are available from the corresponding author upon reasonable request.

## Code availability

Codes for data cleaning and analysis are available from the corresponding author upon reasonable request.
